# COVID-19 omicron variant outbreak in a hematopoietic stem cell transplant unit

**DOI:** 10.1007/s12185-023-03638-3

**Published:** 2023-08-02

**Authors:** Andrea Gilioli, Paola Bresciani, Erica Franceschini, Andrea Messerotti, Valeria Pioli, Corrado Colasante, Francesca Bettelli, Davide Giusti, Fabio Forghieri, Monica Morselli, Elisabetta Colaci, Leonardo Potenza, William Gennari, Monica Pecorari, Roberto Marasca, Anna Candoni, Cristina Mussini, Tommaso Trenti, Patrizia Comoli, Mario Luppi, Angela Cuoghi

**Affiliations:** 1 Section of Hematology, Department of Medical and Surgical Sciences, Azienda Ospedaliero-Universitaria di Modena, University of Modena and Reggio Emilia, Via Del Pozzo, 71, 41124 Modena, MO Italy; 2 Infectious Disease Department, Azienda Ospedaliero-Universitaria di Modena, University of Modena and Reggio Emilia, Modena, Italy; 3 Molecular Microbiology and Virology Unit, Department of Laboratory Medicine and Pathological Anatomy, Azienda Ospedaliero Universitaria di Modena, Modena, Italy; 4 Department of Laboratory Medicine and Pathology, Azienda Unità Sanitaria Locale Di Modena, Modena, Italy; 5 Pediatric Hematology/Oncology Unit and Cell Factory, Istituto di Ricovero e Cura a Carattere Scientifico (IRCCS) Policlinico San Matteo, Pavia, Italy

**Keywords:** SARS-CoV-2, COVID-19, Stem cell transplant

## Abstract

Recommendations and guidelines for management of SARS-COV-2 infection in hematologic patients were developed in the very difficult context of dealing with novel viral variants from one pandemic wave to another, with different susceptibility to available drugs and vaccines. Moreover, the largest SARS-COV-2 case series in patients treated for hematologic malignancies, including stem cell transplant recipients, was published before the Omicron surge, and refers mainly to Alpha and Delta viral variants. These infections had very high mortality, in a period when antivirals and monoclonal antibodies were mostly unavailable. Here, we report for the first time a SARS-COV-2 Omicron variant outbreak inside a Bone Marrow Transplant (BMT) Unit, describing the characteristics, clinical course, and infection outcomes shortly before and shortly after myeloablative transplantation. We detail how infections were treated off-label and managed inside the BMT ward, to guarantee the best possible outcomes while avoiding risks for non-infected inpatients. The positive outcomes observed suggest that it may not be absolutely necessary to obtain SARS-CoV-2 PCR negativity before BMT in hematologic patients after treated infection, in cases with long-term PCR positivity and high-risk hematologic disease.

In the last 3 years, transplant centers worldwide faced the challenges of SARS-CoV-2 pandemics. Here, we report the outcomes of two patients infected with SARS-CoV-2 during inward admission for allogeneic stem cell transplant.

Patient 1, 20-year-old male, was diagnosed with acute B-lymphoblastic leukemia, standard risk, in December 2021. After partial remission with induction chemotherapy, he underwent reinduction, obtaining stable disease. After one cycle of blinatumomab, he reached complete remission with molecular measurable residual disease (MRD) negativity and developed paucisymptomatic SARS-CoV-2 infection. Outpatient treatment with nirmatrelvir/ritonavir for 5 days determined symptom remission and negative antigenic swab after 1week. After a second cycle of blinatumomab, with stable MRD-negativity, pre-transplant sequential HRCT scans depicted improving apical consolidative pattern and a broncho-alveolar lavage (BAL) detected *Aspergillus fumigatus*: secondary prophylaxis with posaconazole was prescribed. FEV1 and DLCO were normal for age and smoking habit. ABO bidirectional incompatible, CMV positive (recipient positive) allogeneic stem cell transplant from HLA9/10 MMUD was planned without further SARS-CoV-2 test according to hospital policy. He underwent myeloablative conditioning with TBI-Cy and CsA-MTX-based GVHD prophylaxis with rabbit anti-T-lymphocyte globulin (ATLG, Grafalon 40 mg/kg). On day -6, thrombocytopenia and significant cough not otherwise explained developed, and nasopharyngeal swab detected SARS-CoV-2 RNA positivity at 27 amplification cycles. Analysis on previous BAL revealed SARS-CoV-2 positivity. Chest X-ray and oxygen saturation were normal. Since myeloablative TBI was concluded, we proceeded with the transplant program. Patient was isolated in zero-pressure room inside BMT-unit, to guarantee both the completion of transplant program and the protection of other patients. Immunodeficiency scoring index (ISI) [1] was 8 (high risk). After consultation with infectious disease team, sotrovimab and remdesivir (200 mg day 1 and then 100 mg/d for total 5 days) were infused on days -5, -4, -3, -2, -1. We monitored infection with weekly swab and RNAemia as in Fig. 1.

**Fig. 1:**
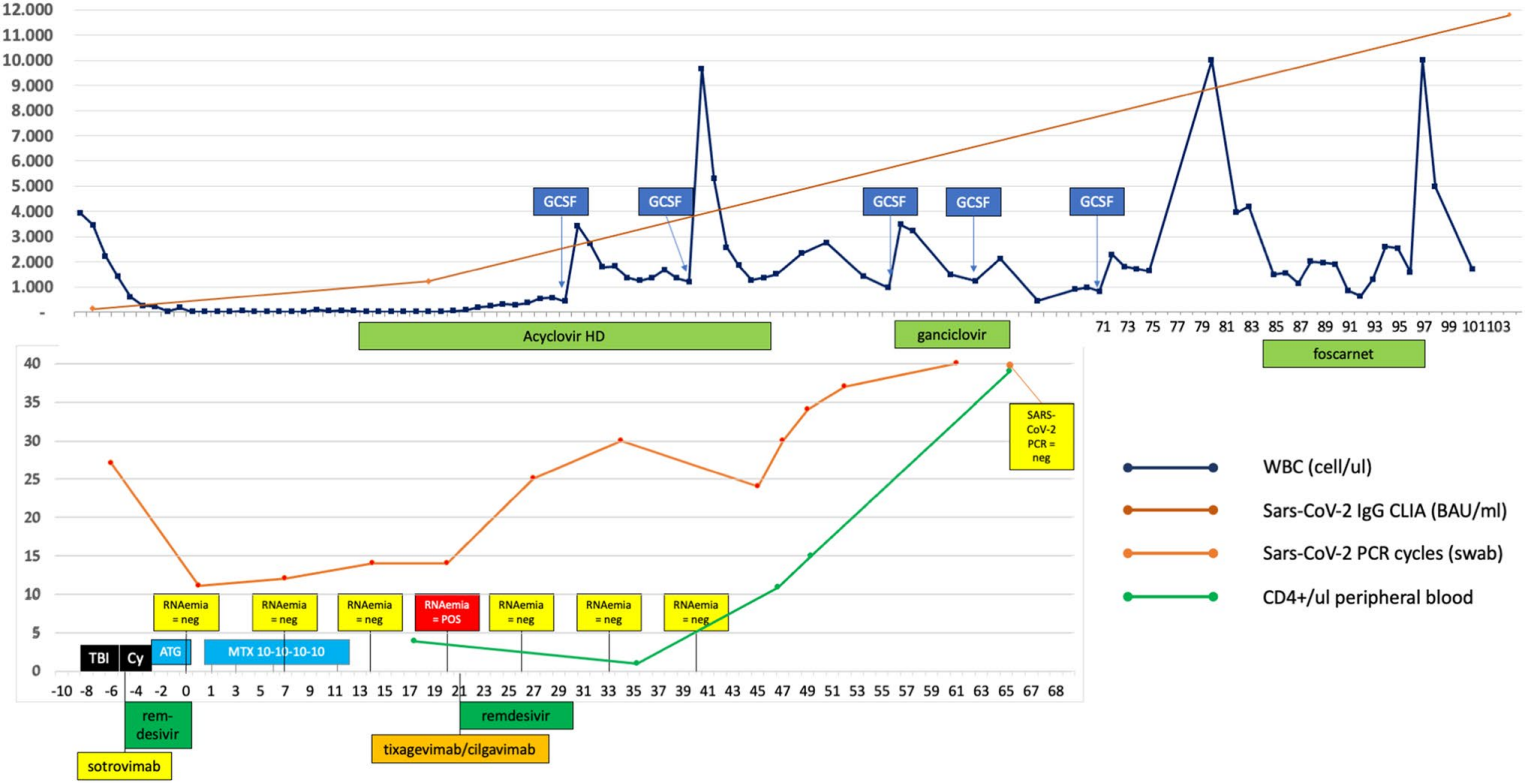
Transplant timeline of patient 1. The x axis represents days before and after transplant, stem cell infusion is on day “0”. In the upper part: white blood cell count (WBC/ul, blue line) and SARS-CoV-2 IgG title (dark orange line), with temporal representation in days or interval of administration of granulocyte-colony stimulating factor (GCSF) and anti-HSV1 and anti-CMV therapies (acyclovir high dose: 10 mg/kg/8 h; ganciclovir: 5 mg/kg/12 h; foscarnet: 90 mg/kg/12 h). In the lower part: SARS-CoV-2 PCR amplification cycles on nasal swab (light orange line), SARS-CoV-2 RNAemia on peripheral blood and CD4 + T cells counts (cells/ul, green line), with temporal representation in days or interval of administration of conditioning therapy, GVHD prophylaxis, antiviral, and monoclonal antibodies used to treat SARS-CoV-2. TBI: total body irradiation (1200 cGy total dose, 200 cGy/fraction). Cy: cyclophosphamide 60 mg/kg/d for 2 days. ATG: anti-T-lymphocyte globulin “Grafalon”, 40 mg/kg total dose. MTX 10-10-10-10: methotrexate 10 mg/sm (days 1, 3, 6, 11). BAU: binding antibody unit

In the meantime, patient 2, 31-year-old female at day + 29 after HLA-identical sibling donor transplant in acute myeloid leukemia NOS, was found SARS-CoV-2 PCR positive at screening swab. Of note, she shared the antechamber filter zone with patient 1. Considering completed neutrophil and platelet engraftment, she was moved to infectious disease ward and her room was left empty. Developing fever, rhinitis, and cough, she was treated with sotrovimab and remdesivir (same schedule as above) and discharged at day + 36. SARS-CoV-2 swab became negative on day + 49. The patient is in complete remission without signs of GVHD at last follow-up on day + 175.

No other patient developed SARS-CoV-2 infection during the following weeks and the scheduled admissions and discharges proceeded as planned.

Patient 1 developed early CTCAE grade 4 mucositis and stomatitis, with HSV1 DNA isolated in oral mucosa and in blood on day + 8. Standard acyclovir prophylaxis (5 mg/kg TID) was switched to therapeutic dosage of 10 mg/kg TID from day + 12, without improvement. On day + 20, SARS-CoV-2 viremia became positive, and stable PCR amplification cycles from nasal swab were found. Patient was afebrile, without oxygen supplementation and with dry cough and stable chest X-rays. Noteworthy, engraftment was not yet reached. Considering susceptibility information on isolated SARS-CoV-2 variant BA5.2.1, a second 10-day course of remdesivir associated with tixagevimab/cilgavimab 300/300 mg was administered. After 1 week, SARS-CoV-2 RNAemia returned negative, while nasopharyngeal swab demonstrated increasing amplification cycles (as in Fig. 1). On day + 28, a single GCSF was given and PMN engraftment was maintained from day + 29, without evidence of engraftment syndrome and with subsequent occasional need for GCSF afterward, as depicted in Fig. 1. After 33 days of high-dose iv acyclovir and persistent significant herpetic stomatitis, with extensive ulcerative lesions in the oral mucosa and positive HSV1 DNAemia, CMV reactivation occurred and iv ganciclovir was given for 10 days; afterward patient could be discharged with mild improving mucositis, and continued valacyclovir 1 g TID at home. SARS-CoV-2 negative PCR swab was reached on day + 66. Platelet engraftment was reached on day + 74. Oral herpetic lesions persisted and increasing HSV1 DNAemia (2393 c/ml at day + 80) was noted, with Pseudomonas infection superimposed; therefore, a short course of parenteral cephalosporins along with foscarnet was given (days + 84–97). After second discharge at day + 99, no further evidence of CMV or HSV1 DNAemia was documented, and oral lesions slowly improved. Patient remains in good clinical condition with MRD-negative complete remission and no signs of GVHD or mucositis at last follow-up on day + 144 were observed.

Recommendations and guidelines [2, 3] to manage SARS-CoV-2 infection for hematologic patients were developed in the difficult context of novel viral variants becoming prevalent from one wave to another, with different susceptibility to drugs and vaccines. The largest SARS-CoV-2 case series in hematologic and transplanted patients [1, 2, 4, 5] was published before Omicron surge and refers mainly to Alpha and Delta variant pandemic waves, carrying high mortality. Besides, at that time, SARS-CoV-2 vaccines, antivirals, and monoclonal antibodies were not available. Noteworthy, these recommendations stress the importance of reaching SARS-CoV-2 molecular negativity before transplant, with AII level of evidence [3]. On the other hand, it is also established that immunocompromised patients may maintain long-term viral shedding [2, 3], and delaying the transplant procedure can carry a high risk of disease relapse. In immunocompetent and mostly immunized population, Omicron variant seems to carry a lower risk of severe disease [6], but data on its impact on hematologic patients have not yet been published. In fact, a recent EBMT survey, based on data acquired up to August 2021, uncovered that the percentage of high-risk hemopoietic cell transplantation deferral in asymptomatic hematological patients long-shedding after COVID-19 was 76.9%, lower than 90.2% in asymptomatic infection without previous COVID-19 [7].

These two cases are interesting for multiple aspects, also in comparison to a previously published case with both stem cell donor and recipient positive for SARS-CoV-2 [8].

Patient 1 had already been treated for SARS-CoV-2 low-risk disease before HSCT, and in absence of clinical suspicion of reinfection/reactivation was not re-screened before commencing conditioning, also due to local recommendations. We decided to maintain the transplant program of this patient after documentation of COVID-19 since myeloablative TBI had already been concluded and delaying the further program would have been detrimental at that point.

Patient 1 was treated off-label with remdesivir for two “cycles” of 5 and 10 days, respectively, in the context of profound immunosuppression and in the context of a very stable respiratory function; nonetheless, he was considered at very high risk of complications for profound immunosuppression, smoking habit, and stable PCR cycles in rhinopharyngeal swabs. Nirmatrelvir/ritonavir, used in the first infection, was not proposed because of the known interaction with CsA and potential risk of reduced absorption with mucositis. On the other hand, sotrovimab was the only monoclonal antibody approved with potential efficacy on Omicron variant. Since approval in therapeutical setting was impending, tixagevimab/cilgavimab was chosen when a second course of therapy was prescribed, to try to boost humoral immunity.

Despite a very early infection, the clinical course of patient 1 and patient 2 was unexpectedly positive, with mild symptoms in absence of lower respiratory tract infection nor need of anti-inflammatory treatment. It can be hypothesized that the profound immunosuppression immediately following HSCT, along with the early use of antiviral therapy, might have hampered the risk of hyper-inflammatory state responsible for the high mortality for some SARS-CoV-2-infected patients [9]. It is not possible to weight the impact of early therapy on the final outcome, but it is reasonable to consider that the prompt use of remdesivir and monoclonal antibodies may have played a role while immune system was likely unable to mount an efficient response.

Moreover, the positive outcomes observed may question the absolute need to obtain SARS-CoV-2 PCR negativity before BMT in high-risk hematologic patients after infection has been treated, in cases of prolonged RNA positivity with high number of PCR amplification cycles.

Finally, we hypothesized that a reactivation with SARS-CoV-2 in such an early peri-transplant period had an impact on HSV1-related stomatitis apparently resistant to high-dose iv acyclovir. Cases of HSV1 reactivation associated with SARS-CoV-2 were already reported [10–12] but to our knowledge, this is the first described coinfection after allogeneic HSCT, with a clinical course for HSV1-related stomatitis much more severe than expected even in the context of myeloablative HSCT with ATG-based GVHD prophylaxis.

## Data Availability

No datasets were generated or analysed during the current study.
